# The Use of Over-the-Counter Sleep Aid Containing Diphenhydramine Hydrochloride Among Saudis

**DOI:** 10.7759/cureus.20622

**Published:** 2021-12-22

**Authors:** Abdullah K Alhwimani, Raed A Aljohani, Bader A Altulaihi

**Affiliations:** 1 College of Medicine, King Saud bin Abdulaziz University for Health Sciences, Riyadh, SAU; 2 Department of Family and Community Medicine, King Abdulaziz Medical City - Ministry of National Guard-Health Affairs, Riyadh, SAU; 3 Department of Family and Community Medicine, King Abdullah International Medical Research Center, Riyadh, SAU

**Keywords:** saudi, insomnia, over-the-counter drugs, diphenhydramine, sleep aid

## Abstract

Background

Diphenhydramine is a first-generation H1 receptor antihistamine that is usually used for the treatment of allergy, insect bites such as mosquitos, bee stings, and some types of skin rashes. However, it possesses antiparkinsonian, antitussive, antiemetic, and most importantly hypnotic properties. This study aimed to assess the prevalence, frequency of use, and dosage of over-the-counter (OTC) sleep aid containing diphenhydramine hydrochloride used among Saudis.

Material and Methods

The study used a descriptive cross-sectional design conducted among the Saudi population. An online self-administered questionnaire was distributed among the selected Saudi nationality using social media platforms. The subjects included were Saudis aged 15 years or older, literate in using social media. The questionnaire included basic demographic characteristics, previous and current history of medication use that contains diphenhydramine hydrochloride, its side effects, and other related behavior of using, such as frequency of use and dosage.

Results

A total of 414 respondents were recruited (51% males and 49% females). Previous and current use of medications that contain diphenhydramine hydrochloride constituted 87.2% and 31.9%, respectively. The most common reason for using sleep aid was insomnia (52.1%). Dizziness or imbalance was the most commonly reported side effect. The Chi-square test revealed that the age group of ≤25 years (p = 0.044), female gender (p = 0.040), being a student (p = 0.015), current use of sleeping aid medications (p < 0.001), and the use of other medications (p < 0.001) were significantly associated with increased use of sleep aid medications.

Conclusion

The excessive use of over-the-counter (OTC) sleep aid medications containing diphenhydramine hydrochloride was widely prevalent among the Saudi population. Younger female students constitute the majority of OTC sleep aid medication users.

## Introduction

Diphenhydramine is a first-generation antihistamine medication that specifically targets H1 receptors. It is usually used for the treatment of allergies, insect bites such as mosquitos, bee stings, and some types of skin rashes. Moreover, it has antiparkinsonian, antitussive, antiemetic, and hypnotic properties. The histaminic receptors can be present in the central nervous system (CNS) and peripheral nervous system (PNS). Diphenhydramine produces sedation by competitively antagonizing the receptors of histamine H1 in the central nervous system. Diphenhydramine use in allergy has been unfavorable in practice because of its sedative property. However, this particular property gave diphenhydramine a whole new purpose of use to be sold in the market as a nonprescription over-the-counter (OTC) sleep aid [1].

It is sold in the Saudi market under different brand names, including Panadol Night, Flutab, Adol PM, and Histop. Those medications are classified as over-the-counter (OTC), which means they neither require a medical prescription nor continuous assessment [2]. Hence, as these OTC remedies can be easily accessible and affordable, they can also be abused or overused. This can lead to harmful side effects and significant drug interactions with other medications, ranging from common to rare in occurrence. The common side effects include sedation or sleepiness, incoordination, attention disturbance, dry mouth, fatigue, headache, and dizziness [3]. In addition, the rare side effects include tremor, convulsions, paresthesia, dyskinesia, vertigo, nausea/vomiting, constipation/diarrhea, dyspepsia, tinnitus, chills, confusion, depression, restlessness, euphoria, anxiety, hallucinations, insomnia, palpitations, hypotension, arrhythmia, blood disorders, hypersensitivity reactions, liver dysfunction, rash, urticaria, dyspnea, urinary retention, dysuria, blurred vision, and anorexia [3].

A study conducted on 1025 participants in the United States reported that 59% of the adult participants had consumed the wrong dose of diphenhydramine to enhance their sleep within the past month [4]. Furthermore, during our literature review, we found only one study conducted in Saudi Arabia, targeting primary healthcare patients only. This study was done on 320 participants and reported that 12.81% of the participants used sleeping pills, reaching the conclusion that the usage of sleeping pills among Saudi adults who visit primary care is not common, and further studies with a larger sample are required [5]. Therefore, we decided to conduct a study to assess the prevalence of usage, frequency, and dosage reported by the users. Moreover, by assessing these variables and documenting the common side effects, our research will help spread awareness about the conscious behavior of using over-the-counter sleep aids. Also, it will give a hint to the healthcare society about how common the use of sleep aid is, the reasons for the usage, and the reported side effects by the users.

## Materials and methods

The study used a descriptive cross-sectional design conducted among the Saudi population. Data were collected from August 2021 to October 2021. Ethical approval was obtained from the Institutional Review Board (IRB) of King Abdullah International Medical Research Center (approval number IRBC/1720/21) on July 22, 2021, and an informed consent letter was obtained from all study participants. The subjects included were Saudis 15 years or older, literate in using social media. The exclusion criteria included the following: non-Saudi, younger than 15 years old, non-Arabic speaking, or unable to access the online platform containing our questionnaire.

An online self-administered questionnaire was uploaded to Google Forms and then distributed among the Saudi population using social media platforms including WhatsApp, Twitter, and Telegram. A pre-validated self-administered questionnaire that was used in a previous study (Aljohani et al., 2019) was utilized for data collection with permission from the first author [5]. Minor modifications were done to the questionnaire to fit our study goal, including changing the questions from sleep medications in general to only the ones containing diphenhydramine hydrochloride. The modified questionnaire was reviewed and approved by King Abdullah International Medical Research Center. The modified questionnaire was in the Arabic language, and from Arabic, the questionnaire was translated to English to assess validity, ensure simplicity, and analyze the data. Prevention of double responses was done by limiting responses to only one response per participant. The modified questionnaire included questions about basic demographic characteristics, previous and current history of medication use that contains diphenhydramine hydrochloride, its side effects, use of other medications, and other questions related to the behavior of using, such as frequency (daily and monthly) and dosage.

Non-probability convenience sampling was used by digitally distributing a questionnaire to all eligible and willing respondents. As per the Saudi General Authority for Statistics, the number of Saudi adults (≥15 years old) was 13,986,153 people in 2016 [6]. Using the Raosoft website’s calculator with a confidence interval of 0.95 and a margin of error of 0.05, the sample size was determined to be 385 individuals. A total of 430 responses were collected to adjust for no/lacking responses, and then, 16 were excluded due to incompletion or illegibility, making the net outcome 414.

The data were analyzed using Statistical Packages for Social Sciences (SPSS) version 26 (IBM Corp., Armonk, NY, USA). Both descriptive and inferential statistics had been conducted. In descriptive statistics, all categorical variables had been presented using numbers and percentages. The previous history of medication use that contains diphenhydramine hydrochloride was compared with the sociodemographic characteristics using the Chi-square test. A p-value cutoff point of 0.05 at 95% CI was used to determine statistical significance. The prevalence of sleep aid use that contains diphenhydramine hydrochloride among the sample was described using numbers and percentages.

## Results

A total of 414 participants were enrolled. Table 1 presents the sociodemographic characteristics of the participants. The most common age group was 17-25 years (55.1%), with more than half being males (51%), and nearly all had bachelor’s or higher degrees (82.9%). Unmarried participants constituted 68.1%, and 47.6% were students. The history of medication use containing diphenhydramine hydrochloride constitutes 87.2%, while the current usage of this type of medication was reported to be 31.9%. About 32% were using other types of medication, and the most common of them were painkillers (21.6%) and antidiabetic medications (17%).

**Table 1:** Participant’s sociodemographic characteristics and the use of medications (n = 414) *Only 88 participants were using other types of medication, which were the subject of the analysis. †Variable with multiple response answers.

Study variables	N (%)
Age group	
17–25 years	228 (55.1%)
26–35 years	69 (16.7%)
36–45 years	46 (11.1%)
>45 years	71 (17.1%)
Gender	
Male	211 (51%)
Female	203 (49%)
Educational level	
High school or below	71 (17.1%)
Bachelor or higher	343 (82.9%)
Marital status	
Unmarried	282 (68.1%)
Married	132 (31.9%)
Occupational status	
Employed	152 (36.7%)
Unemployed	65 (15.7%)
Student	197 (47.6%)
History of medication use that contains diphenhydramine hydrochloride	
Yes	361 (87.2%)
No	53 (12.8%)
Current use of medication that contains diphenhydramine hydrochloride	
Yes	132 (31.9%)
No	282 (68.1%)
Use of other medication	
Yes	88 (21.3%)
No	326 (78.7%)
Specific type of other medication use*^†^	
Painkillers	19 (21.6%)
Antidiabetic medications	15 (17%)
Melatonin	12 (13.6%)
Antihypertensive	11 (12.5%)
Antidepressant	8 (9.1%)
Thyroid medications	6 (6.8%)
Supplements	6 (6.8%)
Statin	5 (5.7%)
Others	15 (17%)

**Figure 1:**
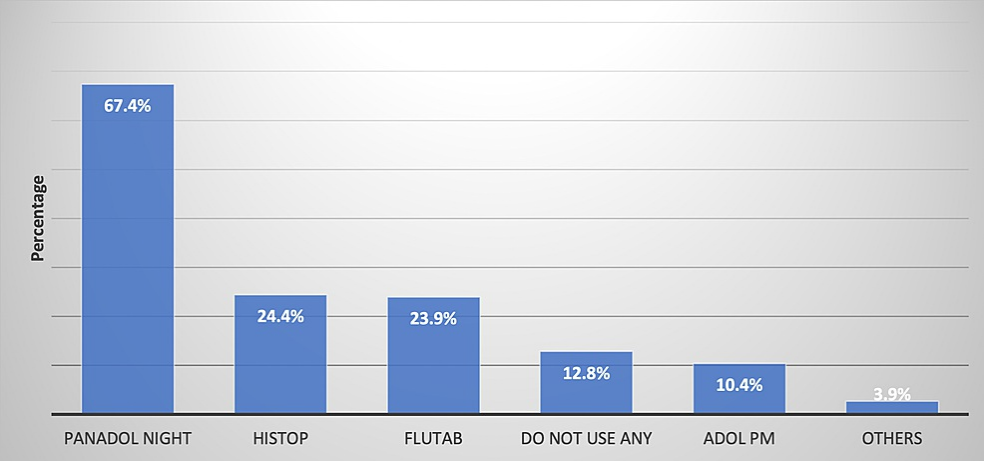
Previous history of medication use that contains diphenhydramine hydrochloride (n = 414)

**Figure 2:**
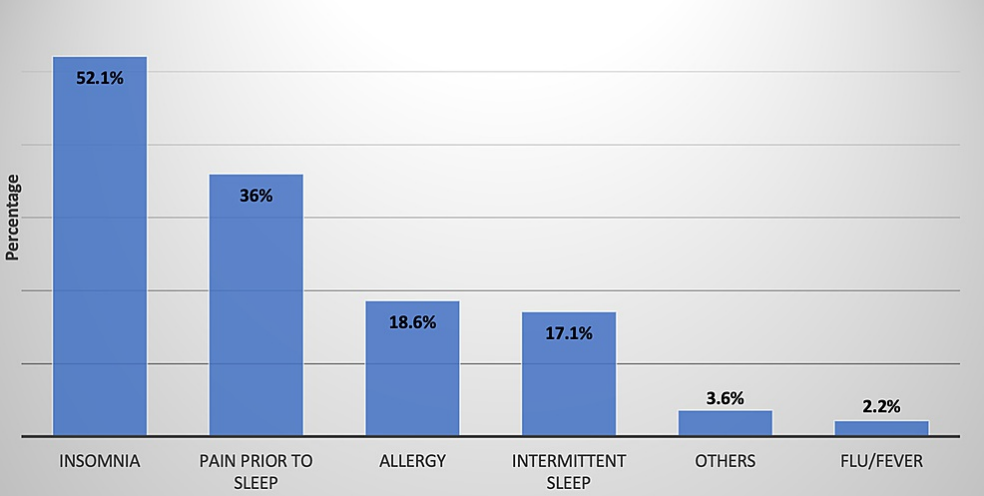
Reasons for using medications that contain diphenhydramine hydrochloride (n = 361)

The most commonly used type of medication was Panadol Night (67.4%), followed by Histop (24.4%) and then Flutab (23.9%) (Figure 1). In Figure 2, the most common reason for using medications that contain diphenhydramine hydrochloride was insomnia (52.1%) and nighttime pain (36%). The behaviors of the participants using medications containing diphenhydramine hydrochloride are given in Table 2. It can be observed that 31.9% were using this type of medication once per day, while 32.1% reported using it on a monthly basis. About 67% of the total population reported satisfactory results when taking one pill only each time needed. Sleep aid medications’ most common side effects are anticholinergic symptoms that include dizziness or imbalance (28%), followed by dryness of the mouth, nose, and throat (19.7%).

**Table 2:** Participants’ behavior regarding the use of medications that contain diphenhydramine hydrochloride (n = 361)* *Fifty-three respondents had no previous history of use of medications that contain diphenhydramine hydrochloride; they were excluded from the analysis. †Variable with multiple response answers.

Characteristics	N (%)
On a daily basis, how frequently do you use over-the-counter sleep medications that contain diphenhydramine hydrochloride?	
Once per day	231 (31.9%)
Twice per day	9 (2.5%)
Thrice per day	1 (0.30%)
One to two times per week	6 (01.7%)
One to two times per month	14 (03.9%)
Rarely	55 (15.2%)
As needed	45 (12.5%)
On a monthly basis, how frequently do you use over-the-counter sleep medications that contain diphenhydramine hydrochloride?	
Once per month	116 (32.1%)
Twice per month	44 (12.2%)
Thrice per month	45 (12.5%)
More than five times per month	8 (2.2%)
A couple of times per week	97 (26.9%)
Rarely	30 (8.3%)
As needed	21 (5.8%)
How many pills do you take at a time	
One pill	242 (67%)
Two pills	110 (30.5%)
Three pills	2 (0.60%)
Other	7 (1.9%)
Side effect of sleep medications^†^	
Dizziness or imbalance	101 (28%)
Dryness of the mouth, nose, or throat	71 (19.7%)
Anxiety, nervousness, or hyperactivity	64 (17.7%)
Palpitations	60 (16.6%)
Constipation or diarrhea	39 (10.8%)
Abdominal pain	34 (09.4%)
Muscle weakness	31 (08.6%)
Visual disturbances	27 (07.5%)
Nausea or vomiting	25 (06.9%)
Skin irritation	9 (2.5%)
Difficulty or painful urination	7 (1.9%)
Others	3 (0.80%)

In Table 3, when measuring the relationship between the previous history of use of medications containing diphenhydramine hydrochloride in regards to the sociodemographic characteristics and their behavior of use, it was found that the prevalence of respondents who had a history of using sleep aid medications was more common among the age group of ≤25 years (p = 0.044), female gender (p = 0.040), those who were currently using it (p < 0.001), and those who were using other types of medications (p < 0.001).

**Table 3:** Relationship between the previous history of use of medications that contain diphenhydramine hydrochloride among the sociodemographic characteristics and their behavior of using it (n = 414) ^§^p-value has been calculated using the Chi-square test. **Significant at p < 0.05 level.

Factor	History of use of medications that contains diphenhydramine hydrochloride		p-value^§^
	Yes (N (%)) ^(n = 361)^	No (N (%)) ^(n = 53)^	
Age group			
≤25 years	192 (53.2%)	36 (67.9%)	0.044 **
>25 years	169 (46.8%)	17 (32.1%)	
Gender			
Male	177 (49%)	34 (64.2%)	0.040 **
Female	184 (51%)	19 (35.8%)	
Educational level			
High school or below	65 (18%)	6 (11.3%)	0.228
Bachelor or higher	296 (82%)	47 (88.7%)	
Marital status			
Unmarried	241 (66.8%)	41 (77.4%)	0.122
Married	120 (33.2%)	12 (22.6%)	
Occupational status			
Employed	140 (38.8%)	12 (22.6%)	0.015 **
Unemployed	59 (16.3%)	6 (11.3%)	
Student	162 (44.9%)	35 (66%)	
Current use of medication that contains diphenhydramine hydrochloride			
Yes	132 (36.6%)	0	<0.001 **
No	229 (63.4%)	53 (100%)	
Use of other medication			
Yes	88 (24.4%)	0	<0.001 **
No	280 (75.6%)	53 (100%)	

## Discussion

This study attempted to examine the use of over-the-counter sleep aid medications containing diphenhydramine hydrochloride among the Saudi population. This study revealed that the previous history of sleep aid medication use among the Saudi population was relatively high, at 87.2%, among which 31.2% reported current use. In a paper published by Aljohani et al., the use of sleeping pills without prescription was found to be low at 12.8% [5]. Another paper conducted in Riyadh, Saudi Arabia, indicated that medical students in King Saud University College of Medicine during the year 2011 had been reported to have been using sedative drugs since enrolment, with 17% of them expressing sedative drug use at some time since enrolment [7]. These findings corroborate the study of Al-Naggar et al., where 7.5% of university students used sleeping pills primarily due to depression or to induce sleep [8]. In our study, the use of sleep aid medications was significantly higher among students, consistent with previous results. The use of sleep aid medications among the young ones can be very alarming, as such Al-Naggar et al. suggested that the education among university students regarding the use of sleeping pills is vital to prevent any adverse reactions regarding the inappropriate usage of this type of medication [8].

In the United States, a study reported that although 19% of the adult population were using sleep aid with prescription, 26.5% of them were using OTC, which was comparable with our results [9]. Similarly, a study by Abraham et al. indicated that about 59% of the older adults had used a potentially inappropriate OTC medication containing diphenhydramine or doxylamine to improve sleep within the past 30 days, which was similarly reported by Agostini et al. [4,10]. In Canada, Vozoris et al. revealed that the overall prevalence of sedative medication use in 2003 was 5.5%, and the rises in its use were notably seen among men, nonelderly, and obese individuals [11]. That conclusion is not consistent with our reports, as the use of OTC sleep aid medication was more prevalent in women than in men. Some papers relate the excessive use of sleep medication with marital status [5,9]. Nevertheless, this is not true in our study, as we found no significant relationship between marital status and the previous use of sleep aid medication.

Our study suggests that insomnia was the most common reason for using sleep aid medications. As more than half (52.1%) of the subjects expressed that the use of OTC sleep aid medication was due to insomnia and nighttime pain (36%). Consistently, Aljohani et al. documented that primary care patients used sleeping pills primarily due to sleeping difficulties and frequent awakenings, while in a paper by Al-Naggar et al., headache (79.5%) and anxiety were the primary and secondary reasons for using sleeping pills, respectively [5,8].

Furthermore, about 31.9% of the respondents were using sleep aid medications daily, and 67% of them were taking one pill at a time, while others were taking two pills at a time. Although the excessive use of sleep aid medications is noticeable, its prolonged use could be detrimental to health and is associated with many health problems, such as motor incoordination, lassitude, slowed reaction times, dysarthria, ataxia, nausea, headache, and drowsiness [12]. In fact, it could be related to mortality cases through multiple causes of death, such as cancer, ischemic heart disease, and stroke [13-15]. Consistently, Agostini et al. documented that patients using diphenhydramine were at an increased risk for any delirium symptoms such as inattention, disorganized speech, and altered consciousness [10]. In our study, the most commonly reported adverse effect was dizziness or imbalance (28%), followed by dryness of the mouth, nose, or throat (19.7%) and anxiety, nervousness, or hyperactivity (17.7%). It is necessary to seek expert advice before taking sleep aid medications, specifically among older adults. For instance, Chui et al. suggested that community pharmacists should advise older adults to consult a physician prior to recommending OTC sleep aid due to concerns in medication safety [16].

Besides sleep aid medication containing diphenhydramine hydrochloride, other types of medications had been used by about 21% of the participants, and the most common of them was painkillers (21.6%), followed by antidiabetic medications (17%), melatonin (13.6%), antihypertensive (12.5%), antidepressant (9.1%), thyroid medications (6.8%), supplements (6.8%), and statin (5.7%). Some of these medications may be one of the reasons why the subjects were unable to sleep or had sleep disturbance, which could lead them to take OTC medications, such as thyroid medications. Others aid sleep but do not contain diphenhydramine hydrochloride, such as melatonin.

## Conclusions

The excessive use of over-the-counter sleep aid medication containing diphenhydramine hydrochloride was widely prevalent among the Saudi population. Younger female students are the significant users of OTC sleep aid medications. Awareness about OTC medications containing diphenhydramine hydrochloride is necessary, especially among students. Moreover, it is better to consult an expert for any signs or symptoms of sleep disorders. Appropriate early intervention and treatment could lead to proper sleep practices. Further research is recommended to validate the high prevalence of OTC medication use containing diphenhydramine hydrochloride in our region.
